# Efficacy and Safety of a Diet Enriched with EPA and DHA, Turmeric Extract and Hydrolysed Collagen in Management of Naturally Occurring Osteoarthritis in Cats: A Prospective, Randomised, Blinded, Placebo- and Time-Controlled Study

**DOI:** 10.3390/ani14223298

**Published:** 2024-11-16

**Authors:** Manuela Lefort-Holguin, Aliénor Delsart, Colombe Otis, Maxim Moreau, Maude Barbeau-Grégoire, Florence Mellet, Vincent Biourge, Bertrand Lussier, Jean-Pierre Pelletier, Johanne Martel-Pelletier, Eric Troncy

**Affiliations:** 1 Groupe de Recherche en Pharmacologie Animale du Québec (GREPAQ), Université de Montréal, Saint-Hyacinthe, QC J2S 2M2, Canada; manuela.lefort-holguin@umontreal.ca (M.L.-H.); alienor.delsart@umontreal.ca (A.D.); colombe.otis@umontreal.ca (C.O.); m.moreau@arthrolab.com (M.M.); maude.barbeau-gregoire@umontreal.ca (M.B.-G.); bertrand.lussier@umontreal.ca (B.L.); 2 Osteoarthritis Research Unit, University of Montreal Hospital Research Center (CRCHUM), Montréal, QC H2X 0A9, Canada; dr@jppelletier.ca (J.-P.P.); jm@martelpelletier.ca (J.M.-P.); 3 Royal-Canin S.A.S., F-30470 Aimargues, France; florence.mellet@royalcanin.com (F.M.); vincent.biourge@royalcanin.com (V.B.)

**Keywords:** omega-3, curcuminoid, feline, chronic pain, biomechanical, functional alterations, degenerative joint disease

## Abstract

This study evaluated a therapeutic diet high in eicosapentaenoic (EPA) and docosahexaenoic acid (DHA) of marine source (EPA:DHA ratio 0.69:1), turmeric extract, and hydrolysed collagen in cats (*N* = 30) with naturally occurring osteoarthritis (OA) over a 13-week (W) period, followed by a 4-W washout, using four previously validated pain/functional outcomes. Compared to the placebo diet, the therapeutic diet significantly improved peak vertical force (*p* = 0.017; W16, 64% responders), correlating to enhanced weight bearing; stair assay compliance (*p* < 0.001; W16, 87% responders), reflecting reduced fatigue related to OA pain; night-time actimetry (cohort effect; *p* = 0.05, 67% responders), suggesting greater spontaneous mobility and comfort; and MI-CAT(V) score (cohort effect; *p* < 0.001, 80% responders), indicating reduced functional impairments. The earliest therapeutic response was observed at W06, marked by an inflection point between actimetric linear regressions of both cohorts, confirmed by significant MI-CAT(V) differences (*p* < 0.007; W08; W12; W16). The MI-CAT(V) clinical metrology instrument was validated for inter-rater reliability, minimal placebo effect (<15% responders), and OA severity clustering. Despite baseline differences in biomechanical assessments (*p* < 0.05), both moderate and severe OA clusters responded equally positively to the therapeutic diet. Based on all outcomes, the therapeutic diet showed promise for the long-term management of feline OA, with no observed side effects.

## Simple Summary

Feline osteoarthritis (OA) is a painful degenerative musculoskeletal disease characterised by functional alterations and degraded animal welfare. As OA cannot be cured, veterinarians’ therapeutic goal is to reduce pain symptoms to better the animal’s quality of life. The pharmacological arsenal is reduced in cats because of the specific therapeutic margin and the occurrence of side effects. Non-pharmacological avenues such as nutraceuticals are promising. However, there is a need for strong, evidence-based research on the effects of nutraceuticals in feline OA. The present study evaluated the efficacy and safety of a therapeutic diet containing omega-3s, hydrolysed collagen and turmeric extract with a clinical metrology instrument and three objective outcomes that were all previously validated as specific, sensitive and reliable. The diet was proven to provide a clear treatment effect in all cats, including those who were moderately and severely affected, to yield a high rate of compliance to administration and to be devoid of any side effect, which is of fundamental importance when treating chronic illnesses like OA.

## 1. Introduction

Osteoarthritis (OA) is a painful degenerative musculoskeletal disease characterised by joint structural degradation. In cats, OA is primarily caused by the wear and tear of synovial joints, mainly the elbow, hip and stifle joints; therefore, it is strongly correlated with age. Other risk factors include obesity, genetics, and joint trauma (secondary OA), but most have been extrapolated from other species without strong justification [1,2]. Based on the epidemiology studies available to date, 25.6% of cats (*N* = 454/1772, aged 0.2 to 20 years) showed radiographic evidence of OA in at least one appendicular joint [1,3,4,5,6,7,8]. Despite the elevated prevalence of feline OA, the low detection rate from the owner limits the veterinary OA diagnosis and management.

As OA cannot be cured, practitioner’s therapeutic goal is limited to reduce pain symptoms and improve biomechanical function to better the patient’s quality of life [9]. Several strategies can be implemented, alone or in conjunction, pharmaceutical or non-pharmacological. The main pharmacological therapies are non-steroidal anti-inflammatory drugs (NSAIDs) and immunotherapy, although other drugs such as gabapentin and tramadol are also prescribed complementarily. The NSAIDs were the most recommended drugs based on their efficacy despite their known side effects such as gastrointestinal irritation, nephrotoxicity and hepatotoxicity, that limit their long-term use. Immunotherapy, and specifically the novel anti-nerve growth factor monoclonal antibody frunevetmab (Solensia^®^, Zoetis, Inc., Parsippany, NJ, USA), opens up new perspectives against chronic OA pain in cats. However, information to support individual case recommendation and the best use in long-term treatment are lacking, including safe combinations with other drug(s), NSAID particularly, and the possibility of inducing adverse effects during repeated administration over a very long period (5 to 8 years). There are various non-pharmacological strategies, amongst them are nutraceuticals. From this category, there is evidence of effective OA management with omega-3-, collagen- and plant-based approaches [10]. Omega-3 long-chain fatty acids like eicosapentaenoic acid (EPA) and docosahexaenoic acid (DHA) can modulate the inflammatory response by promoting some “anti-inflammatory” actions (competition with arachidonic acid enzymes, production of less potent mediators) and stimulate specialised pro-resolving mediators, thereby instigating endogenous resolution of (neuro)inflammatory response [11,12,13]. Marine oil and green-lipped mussels contain high levels of EPA and DHA and have been reported to have high efficacy for OA management in cats [14]. Collagen-derived dietary supplements have been found to aid in OA management in dogs [15,16,17,18,19,20,21,22,23,24]. However, in their systematic review, Barbeau-Grégoire et al. concluded that a clear determination of collagen efficacy based on the existing results was impossible due to issues in the studies’ methodologies [10]. As for plant-based supplements, turmeric (*Curcuma longa*) is a perennial plant known for its therapeutic properties. Its rhizome contains compounds called curcuminoids, including curcumin, that have been reported to have an important anti-inflammatory effect in OA dogs [25,26]. Nonetheless, very few studies have been conducted on OA cats. This is very unfortunate because when palatable, an effective therapeutic diet yields a high rate of compliance, which is of fundamental importance when treating chronic illnesses like OA.

The aim of this prospective, randomised, blinded, placebo-controlled study was to evaluate the efficacy and safety of a therapeutic diet containing a high level of EPA and DHA, with a specific EPA:DHA (0.69:1) ratio, turmeric extract and hydrolysed collagen in cats with naturally occurring OA.

The four assessment outcomes used in this study were previously validated, demonstrated to differentiate between healthy and diseased cats (specificity) [27,28,29], as well as to detect treatment effects, such as those provided by NSAID (meloxicam or firocoxib), gabapentin, and tramadol (sensitivity) [27,29,30,31,32,33]. Finally, to test the safety of the therapeutic diet on health status, clinical exams and bioanalyses were conducted on a regular basis. The hypothesis was that the enriched therapeutic diet would be superior to the placebo diet on several, if not all, outcomes, over 13 weeks of oral administration.

## 2. Materials and Methods

### 2.1. Ethical Approval

This study was approved by the Institutional Animal Care and Use Committee of ArthroLab, Inc., Montreal, QC, Canada (A194-RCA21F), Université de Montréal (22-Rech-1832) and Royal Canin, S.A.S., Aimargues, France (ERC-200721-31) and was performed between January 2022 and July 2022 at ArthroLab. This research protocol adhered to the Canadian Council on Animal Care guidelines on laboratory animal facilities and US Dept. of Agriculture Animal Welfare Act (9 CFR Parts 1–3).

### 2.2. Inclusion Criteria

Two cohorts of *N* = 15 cats from the ArthroLab colony were selected according to age, physical examination (body weight (BW), body condition score (BCS), health observations), clinical pathological findings (complete blood count (CBC), serum chemistries, and urinalysis), behaviour, group sociability and acclimation performance. Adult senior cats, 7 years and older, had to show radiographic evidence of naturally occurring OA in appendicular joints, as previously described, as well as for inclusion/exclusion criteria [27,28,30,34]. Signs of OA pain were confirmed by rating of the Montreal Instrument for Cat Arthritis Testing, for Veterinarians [MI-CAT(V)] ≥ 9% of alteration score from three evaluators (A.D., M.B.-G. and E.T.), as recently validated for its discriminatory ability [33].

Cats with chronic kidney disease (CKD) up to and including International Renal Interest Society (IRIS) stage 1 were eligible to enrol, provided the disease was stable. An acclimation protocol was performed to ensure every candidate would comply with housing environment and methods of assessment.

### 2.3. Animal Housing

The cats selected for this study and their characteristics (cohort, identification (ID) number, sex, age, BW, number of joints affected, radiographic score, initial MI-CAT(V) score and BCS) are presented in Appendix S1. They were group-housed in lighting-, temperature-, and humidity-controlled rooms. A supply of fresh tap water was available *ad libitum*, and food was provided twice daily (morning and afternoon). Cats were weighed every week to ensure stable BW based on the manufacturer’s recommendations.

### 2.4. Experimental and Control Diets

The diets were a complete and balanced dry food for adult cats, formulated to meet the nutritional levels established by the Association of American Feed Control Officials. Both diets were supplied by the manufacturer, in neutral bags, identified by a code name and were isocaloric. Their composition is detailed in Table 1. The only difference compared to the control (ctrl.) diet was that the experimental (expl.) therapeutic diet contained the following active ingredients: a mix of marine oil, hydrolysed collagen and turmeric extract. Marine oil mix was composed of a combination of fish oil and algal oil which confers to the diet a specific ratio of EPA:DHA (0.69:1). Curcuma extract was associated with a phosphatidylcholine complex and contained approximately 18% of curcuminoids of which 15% was curcumin, 2.8% was demethoxycurcumin and 0.35% was bisdemethoxycurcumin. Hydrolysed collagen was a mix of peptides of which glycine and proline represented more than 35% of the total amino acids content.

### 2.5. Study Design

This study was structured in four successive phases: an acclimation/training phase beginning six weeks before the first day (D)00 of both diets administration; a baseline phase (BSL; W-03 to W-01); a treatment phase (Tx; W00 to W12); and a recovery phase (Ry; W13 to W16) (Table 2). During the acclimation phase, the placebo-ctrl. diet was introduced progressively over 10 days to transition from the pre-existing diet, before providing exclusive feeding with the placebo-ctrl. diet. During the Tx and the Ry phases, two transitions (over 4 days) were operated: From the placebo-ctrl. to the expl. therapeutic diet (from D00 to D03), and inversely from the therapeutic to the placebo-ctrl. diet (from D88 to D91), respectively. The placebo-ctrl. group was fed with the placebo diet over the four successive phases of this study, mimicking same transitions to keep blinding.

### 2.6. Clinical Bioanalyses and Physical Examination

Blood and urine samples were collected during the acclimation, Tx and Ry phases in order to monitor basic parameters in CBC analysis, blood chemistry, and urinalysis (when needed).

### 2.7. Podobarometric Gait Analysis (PGA)

Gait analysis was performed using a pressure-sensitive walkway mat (Strideway HRSW2^®^ System, Tekscan, Inc., Boston, MA, USA), as previously described [27,28,30,34,35,36,37].

Briefly, three valid trials were retained, at each timepoint, for each cat on a maximum of 10 attempts on the walkway mattress. Among kinetic parameters generated, only the maximal loading (i.e., peak vertical force; PVF) was used, being averaged for paw strikes and valid assays at each timepoint. The coefficient of variation (CV) was used to exclude individuals with elevated (CV > 12%) test-to-test variability between trials, and particularly both BSL values.

The limb that yielded the lowest average PVF value in BSL was determined for each cat and defined as the most affected limb [27,28,30,35]. To reduce data variability, PVF values were normalised according to the stance time duration and using BW as covariate. Stance time duration has been used as surrogate for “limb velocity” because it reflects changes in velocity, and ground reaction forces [35,36,37,38,39]. Finally, the rates of individuals improving their most affected limb PVF values (responders to treatment > to BSL) were compared in each cohort of cats.

### 2.8. Stairs Assay Compliance (Stairs)

The Stairs was performed using a staircase of 16 steps (ceramic tiles, step height of 20 cm), as recently published [32,33]. During a four-minute period, cats were encouraged to do the maximum number of up and down steps. The total number of stairs climbed up and down, and the time (seconds) to ascend and descend the staircase were recorded for each passage during the four-minute period. The cohort-summated number of stairs up and down was restrained to all passages where the median of counts for the cohort was equal to 32 (meaning a whole passage up and down of the cohort), thus giving the assurance of a valid representation of the whole cohort (not biased by some outlier cat(s) in each cohort). As previously described [32], to decrease the inter-individual dispersion, the number of stairs, and the time to go up, was normalised with the chest to croup length, and the BW, respectively. The rates of individuals improving their total number of stairs climbed up and down (responders to treatment > to BSL) were compared in each cohort of cats.

### 2.9. Night-Time Actimetry Monitoring (NAM)

The actimetry (or locomotor activity monitoring) was evaluated using a collar-attached tridimensional accelerometer-based activity sensor (Actiwatch-Mini, Minimitter/Respironics, distributed by Bio-Lynx Scientific Equipment, Inc., Montreal, QC, Canada) maintained in place from W-06 to W16. The first three weeks were discarded as considered an adaptation time for the cats to wear the collar. The device was set at local time and configured to create 1 count value per two minutes (=epoch). The amplitude of each count was subsequently translated to a numeric value (from 0 to infinity) referring to the intensity count of actimetry. To exclude periods where human activity and handling interfered with the cats’ activity, only 3 days per week (Friday, Saturday, Sunday) between 5:00 PM to 6:58 AM were considered for the analyses and later referred to as weekends (WEs). NAM total sum was expressed as the group-average total intensity counts for a given WE. Basal condition was assessed for the first three WEs (WE-03, WE-02 and WE-01); the next 13 WE of the Tx phase were recorded (WE00 to WE12), followed by the Ry phase (WE13 to WE16).

This night-time sequence chosen was supported by previous internal study data as well as published data in the last two decades by the GREPAQ group either in dogs or in cats, in laboratory or home conditions, in acute or chronic pain situation [27,28,29,30,31,33,34,40,41,42,43,44,45,46,47,48]. When observed, all adverse technical events, with respect to the accelerometer or accelerometer data, were recorded and excluded. Based on previous experience in NAM assessment in natural OA dogs and cats [27,28,29,30,31,33,34,42,43,44,45], the typical response over time in animals receiving a placebo is a progressive and slow decrease in NAM intensity. Therefore, non-responders were defined as OA cats presenting a decreasing slope in NAM over time, whereas responder presented a stable or increasing slope in NAM [33].

### 2.10. The Montreal Instrument for Cat Arthritis Testing, for Veterinarians [MI-CAT(V)]

Pain was assessed using the validated MI-CAT(V) scale (see Appendix S2 with a Manual of use) [31,33,47]. It includes four items assessment: (1) body posture; (2) gait; (3) obstacles; and (4) global distance examination.

The assessors (A.D., Ph.D. candidate, M.B.-G., MSc candidate and Pr. E.T.) were blinded to the period and group, and experimental design. Two observers (A.D. and M.B.-G.) were present at all timepoints, except W12 for A.D. (sickness). However, E.T. could only assess BSL1, W8 Tx and W16 Ry. The inter-rater reliability, consistency and reproducibility were tested in order to determine the subsequent use of the three observers average, or only one (or two) observer values. The MI-CAT(V) is considered as an alteration score, meaning higher the value, more impaired is the cat. A responder threshold was validated as a decrease in MI-CAT(V) by more than 15% compared to the BSL median value [33].

### 2.11. Clusters of OA Severity

Lastly, cats were sorted into four OA severity clusters based on BSL MI-CAT(V) scores: absent (<9%), mild (9–20%), moderate (21–35%) and severe (>35%) as described by Delsart et al. [33]. Clusters present in this OA cat population were compared at BSL. Subsequent analyses were performed on all outcomes [PGA, Stairs, NAM and MI-CAT(V)] to compare Tx response across OA severity clusters in cohort 1 (expl.).

### 2.12. Statistical Analysis

The statistical analyses were performed using SPSS software (IBM Corp. released 2011, IBM SPSS Statistics for Windows, version 20.0, Armonk, NY, USA). General descriptive statistics [mean (standard deviation), ± standard error of the mean, median, maximum and minimum] were provided and data distribution determined with Shapiro–Wilk test for Gaussian distribution. CV, intra-class correlation coefficient (ICC) estimates and their 95% confidence intervals [95% CI] were calculated using SPSS statistical package, based on a mean-rating (k = 2), absolute-agreement, 2-way mixed-effects model. The ICC index was interpreted as follows, ≤0.50 poor, >0.50 moderate, >0.75 good and >0.90 excellent reliability [49].

The proposed design used repeated measures (BSL, Tx and Ry) by applying linear mixed model or generalized linear mixed model method, depending on data distribution. For each model, homogeneity of variances was tested, and the best structure of the covariance was assessed using information criteria that measure the relative fit of competing covariance model, including testing for any potential effect of cohort, time and their interaction. Responder rates in each cohort were compared with a Fisher’s test, for each outcome. Data were analysed with an alpha value set at 5%. Inferential analysis is detailed for each outcome, when different.

Sub-analyses were conducted when clustering the OA cat population of cohort 1 (expl. therapeutic diet) and 2 (placebo-ctrl. diet). Cluster validation was performed to corroborate the presence of BSL differences across OA severity clusters for all outcomes using one-sided non-parametric tests, the hypothesis being that severe OA would present more alterations than moderate and/or mild OA. Finally, hindlimb PVF, Stairs, NAM and MI-CAT(V), were analysed with time*cohort*cluster mixed model analyses, to categorise OA severity cluster responsiveness to therapeutic diet in cohort 1 (expl.).

The person administering the diets as well as the people conducting the functional tests were blinded to the diet contents. The statistics were conducted in blind conditions.

## 3. Results

### 3.1. Safety and Diet Palatability

Thirty (30) cats [cohort 1, expl. therapeutic diet, *N* = 15; cohort 2, placebo-ctrl. diet, *N* = 15] were included. All cats did not have any abnormalities of clinical importance according to bioanalyses (blood chemistry, CBC analysis and possible urinalysis) and thorough veterinary examinations. At the exception of OA (only or predominantly to hindlimbs), cats were did not have any musculoskeletal or neuronal abnormality. Some cats were known for chronic concomitant conditions that were controlled at the time of this study: Four cats were diagnosed with CKD-IRIS 1 at BSL, two cats in each cohort (expl. and ctrl.). However, one cat from cohort 2 (ctrl.) (Cat ID: F-046) made an evolution to CKD-IRIS 2 and was excluded from this study at week (W)-01. The three other cats remained at same level or even improved their creatinine levels during this study. This expl. therapeutic diet did not aggravate the pre-existing renal condition. A second cat from cohort 2 (ctrl.), ID: F-028, was euthanatised at W15 due to an evolutive oral neoplastic mass.

In consequence, the placebo-ctrl. group included *N* = 14 cats for the statistical analyses (with the exclusion of ID: F-046) and even *N* = 13 for the W16 timepoint (with the added exclusion of ID: F-028), whereas the expl. diet group included *N* = 15 cats. Both groups were equally represented for gender, BW, radiographic scoring, as well as the initial MI-CAT(V) scores (Table 3). Both diets were observed to be palatable and appetent during the whole follow-up, and BW stable over both groups.

### 3.2. PGA

The BSL reliability was good (ICC = 0.757 [95% CI 0.708–0.799]) and as a result, the mean BSL was used in the following analyses. The only exception in individual values was for the cat ID: F-021 (cohort 1, expl.) who presented a high CV (28.9%) for the PVF whereas the average CV was 5.3% for cohort 1 (expl.; F-021 excluded) and 5.6% for cohort 2 (ctrl.). For this reason, F-021 was excluded from the subsequent inferential analysis. Shapiro–Wilk and Levene’s tests showed that both cohorts were normally distributed (*p* > 0.077) and that the variances were homogenous (*p* = 0.321) for the most affected (hind)limb PVF data normalised by stance time duration. All BSL lowest values affect hind limbs, recognised as most affected limbs.

**Figure 1:**
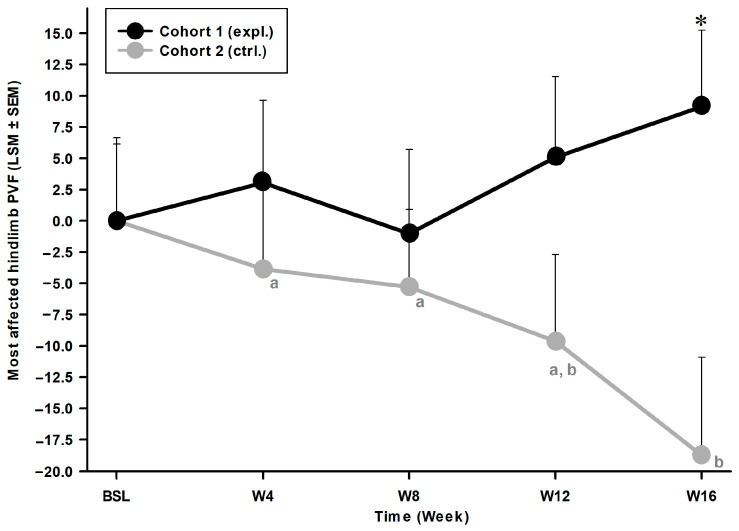
Least square mean (LSM) with positive standard error of mean (SEM) of the most affected hindlimb PVF values normalised by stance time duration using BW as a covariate. Peak vertical force (PVF) recorded for the most affected hindlimb over time for cohort 1 [experimental (expl.) therapeutic diet] and 2 [placebo-control (ctrl.) diet]. Data are expressed relatively to BSL (BSL as 0% for both cohorts). Graph shows an increase in PVF values for cohort 1, suggesting lameness improvement. For cohort 2, graph shows decreasing PVF values, indicating lameness deterioration. * Significant inter-cohort difference (*p* < 0.05; see text for exact *p*-value). a, b Within-time difference. When considering two timepoints, different letters indicate a significant difference (*p* < 0.05; see text for exact *p*-values).

The most affected hindlimb PVF values showed a significant time*cohort effect (F (4, 103.500) = 3.805; *p* = 0.006). Cohort 2 (ctrl.) PVF values decreased with time (Figure 1), indicative of a progressive and continuous deterioration in their biomechanical condition. At W16 [374.75 (83.98)%BW/s], the PVF values from the placebo group (cohort 2) were significantly lower than the ones at average BSL [443.95 (101.11)%BW/s; *p* = 0.001], at W04 [429.32 (138.78)%BW/s; *p* = 0.009] and W08 [430.16 (92.04)%BW/s, *p* = 0.021]. The worsening condition in cohort 2 (ctrl.) reached a significant intergroup difference at W16 (*p* = 0.017), compared to cohort 1 (expl.) which showed a continuous, but statistically non-significant, increase with time.

The inter-cohort difference in responder rate (Table 4) was significant for the most affected hindlimb PVF at W16 (*p* < 0.001) with 64% of cats responding to Tx in cohort 1 (expl.) and 15% of cats showing a placebo effect in cohort 2 (*p* = 0.006). A maximal delta intensity of 26% was observed at W16 compared to cohort 2 (ctrl.). Overall, cohort 1 (expl.) showed a 10% increase in the most affected hindlimb PVF values (or decreased lameness) during Ry, and this was confirmed by a higher responder rate in cohort 1 (expl.) at W16, compared to cohort 2 (ctrl.).

### 3.3. Stairs

Because of the non-Gaussian distribution of total number of stairs climbed up and down (*p* < 0.002) and the time to climb up per passage (*p* < 0.009) in both cohorts, and the BSL mean value for cohort 1 (expl.), 145 steps, different from cohort 2 (ctrl.), 205 steps (*p* = 0.007), a relative representation (BSL as 0% for both cohorts) was adopted.

Cohort 1 (expl.) climbed more steps both during Tx (57% increase at W12; *p* = 0.010) and Ry (82% at W16; *p* < 0.001) compared to the nadir of steps observed at W04, when cohort 2 (ctrl.) did not change with time.

**Figure 2:**
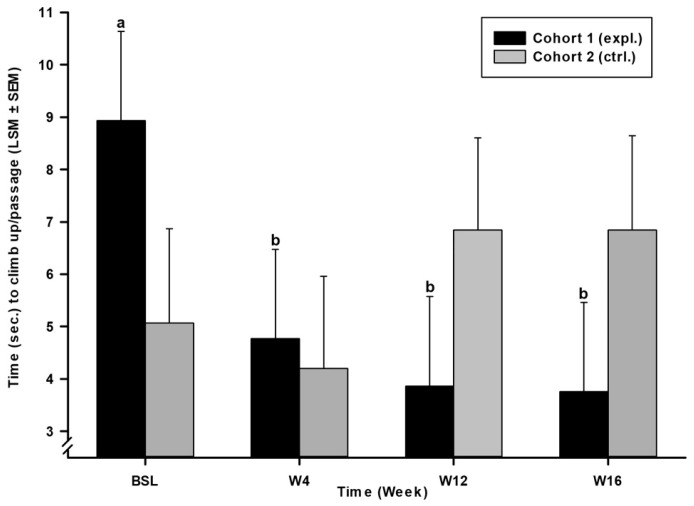
Time (seconds) to climb up per passage, over time for cohort 1 (expl.) and 2 (ctrl.). The histogram shows a decrease in average time required to complete one passage up the 16 stairs for cohort 1 over time, while cohort 2 (ctrl.) remained stable. a, b Within-time difference. When considering two timepoints, different letters indicate a significant difference (*p* < 0.05; see text for exact *p*-values).

For the time to climb up at each passage (Figure 2), compared to BSL [8.93 (11.41) s.], cohort 1 (expl.) performed faster both during Tx at W04 [4.77 (6.10) s.; *p* = 0.021), W12 [3.86 (2.67) s.; *p* = 0.006), and during Ry at W16 [3.76 (2.68) s.; *p* = 0.005). Cohort 2 (ctrl.) remained statistically stable over time (F (3, 78.198) = 1.492; *p* = 0.223).

**Figure 3:**
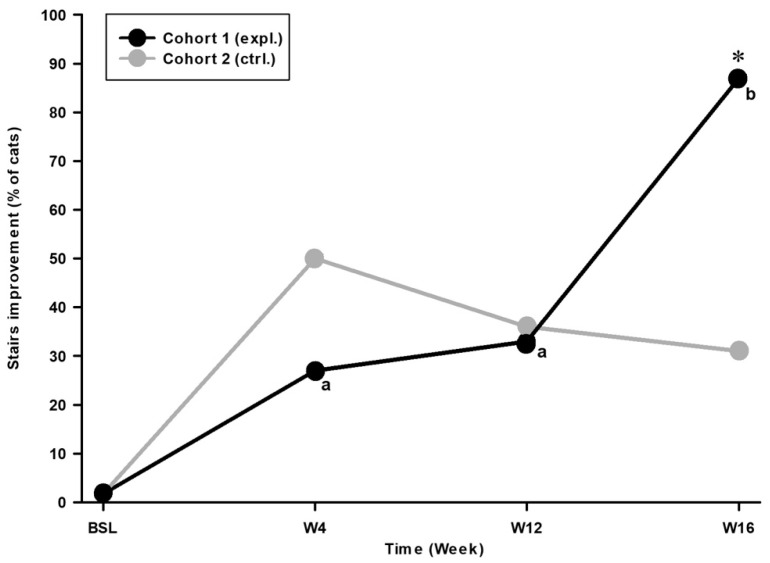
Stairs assay compliance (Stairs) improvement over time for cohort 1 (expl.) and 2 (ctrl.). Graph shows increasing numbers of cohort 1 responder cats improving their total number of stairs climbed up and down, over time, while cohort 2 remained relatively stable. * Significant inter-cohort difference (*p* < 0.05; see text for exact *p*-value). a, b Within-time difference. When considering two timepoints, different letters indicate a significant difference (*p* < 0.05; see text for exact *p*-values).

For the percentage of responder cats in each cohort, the analysis was performed using a binary logistic regression (1 = the cat improved its performance; 0 = the cat did not improve its performance). The time*cohort effect was statistically significant (Wald Khi2 = 9.056; *p* = 0.011), with the placebo group (cohort 2) being quite stable over time (Figure 3). The percentage of improving cats (Table 4) in cohort 1 (expl.) increased significantly at Ry (87% responders at W16) when compared to the Tx period (27% responders at W04, and 33% responders at W12; *p* < 0.001). The inter-cohort difference was significant at W16 (Figure 3) signifying cohort 1 87% responders showed less OA-related fatigue during Ry, compared to cohort 2 (31%; *p* < 0.001). The maximal intensity of difference between both cohorts of 42% was observed at W16, also with a 78% increase in activity for cohort 1 (expl.), compared to BSL.

### 3.4. NAM

During data collection, one cat, ID: F-017, in cohort 2 (ctrl.) had numerous actimetry collar technical malfunctions, thus he was excluded from the NAM analysis (cohort 2 (ctrl.), N = 13). Cat ID: F-028, euthanatised at W15 was conserved up to WE14. Both cohorts 1 and 2 presented reproducible NAM for the three WEs of BSL recording (ICC = 0.841 [95% CI 0.728–0.917]). However, these BSL were different from each other cohort. The difference of data distribution was confirmed with Shapiro–Wilk normality testing. The cohort 2 (ctrl.) data presented a Gaussian distribution (*p* = 0.070), but not the cohort 1 data (expl.) (*p* = 0.020). Moreover, at BSL, the Levene homogeneity of variance test based on the median was significant (*p* < 0.002), confirming the two groups were not homogenous.

**Figure 4:**
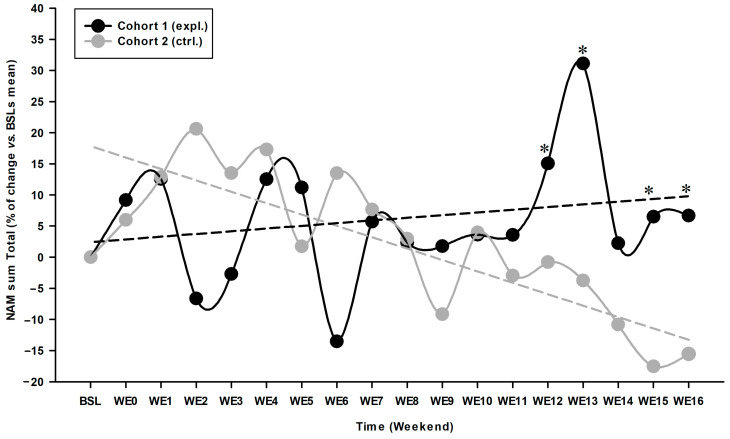
Total night-time actimetry monitoring (NAM) evolution vs. baseline (BSL) average. NAM was continuously recorded over 20 WEs: Three WEs of BSL and 13 WEs (W00 to W12) of recording after initiating the experimental (expl.) diet (cohort 1) or placebo-control (ctrl.) diet (cohort 2), completed by a four WE recovery period (WE13 to WE16) for both cohorts. Linear regression shows increasing mobility for cohort 1 (expl.) and decreasing mobility for cohort 2 (ctrl.) over time, with an inflection point between both linear regressions around WE06. NAM sum total (mean) was expressed in percentage (%) of change vs. BSL average for both cohorts (BSL as 0% for both cohorts). * Significant inter-cohort difference (*p* < 0.05; see text for exact *p*-values for intra- and inter-cohort differences).

For NAM, type III effects for cohort (F (1, 34.127) = 3.935; *p* = 0.05) and time*cohort (F (19, 404.658) = 1.878; *p* = 0.014) were significant, highlighting a difference of evolution between both cohorts. Intra-group NAM evolution (Figure 4) revealed for cohort 1 (expl.) an increase in motor activity between WE07 and WE16, supported by a significant intergroup difference at WE12 (*p* = 0.050; 15.85%), WE13 (*p* = 0.009; 34.86%), WE15 (*p* = 0.041; 24.06%) and WE16 (*p* = 0.037; 22.24%), when compared to cohort 2 (ctrl.), the latter presenting a clear declining slope over time.

Finally, the individuals’ distribution as responders (=ascending or null slope of evolution in NAM) or non-responders (=descending slope) confirmed the improvement observed in cohort 1 (expl.), where most OA cats (67%) improved their condition (Table 4), whereas a majority of cats (77%) in cohort 2 (ctrl.) deteriorated in their mobility over the same period (*p* = 0.030). The maximal intensity of difference between cohorts of 35% was observed at WE13, with a 31% increase in activity for cohort 1 (expl.), compared to BSL.

### 3.5. MI-CAT(V)

For BSL 01, the ICC between the three raters was moderate (ICC = 0.588 [95% CI 0.379–0.761]) and for BSL 02, the ICC between A.DE. and M.B.-G. was good (ICC = 0.817 [95% CI 0.607–0.915]). Thus, the evaluators mean was used to calculate the reliability between BSL 01 and 02. The test–retest reliability was also good (ICC = 0.893 [95% CI 0.786–0.948]) and as a result, the mean BSL was used in the following descriptive and inferential analyses. Data were normally distributed into both cohorts (*p* > 0.264) and homoscedastic (*p* = 0.810).

**Figure 5:**
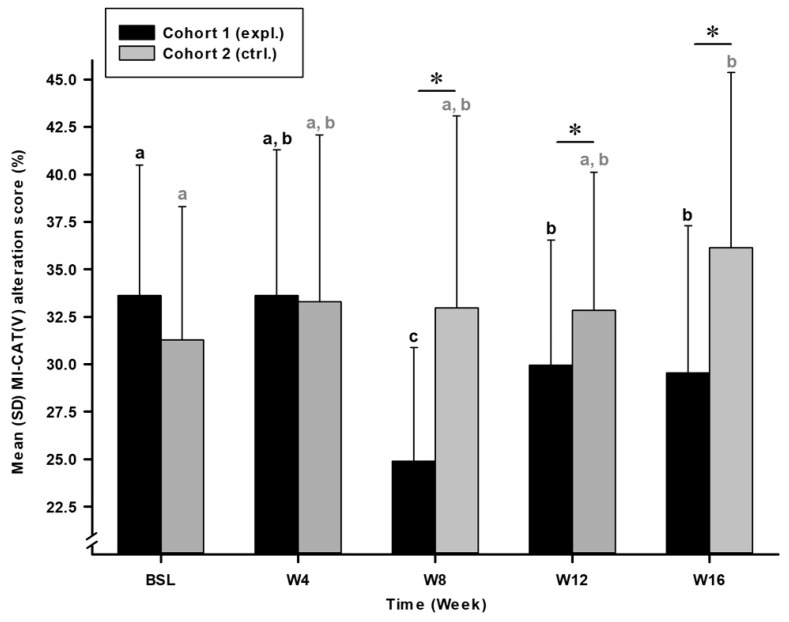
Montreal Instrument for Cat Arthritis Testing, for Veterinarians [MI-CAT(V)] alteration score (%) over time for cohort 1 (expl.) and 2 (ctrl.). Data were presented as the mean (SD). Histogram shows osteoarthritis (OA)-associated biomechanical alterations variable for cohort 1 (expl.) and increasing over time for cohort 2 (ctrl.). * Significant inter-cohort difference (*p* < 0.05; see text for exact *p*-values). a, b, c Within-time difference. When considering two timepoints, different letters indicate a significant difference (*p* < 0.05; see text for exact *p*-values).

The total MI-CAT(V) score (Figure 5) expressed significant time (F (4, 42.122) = 4.784; *p* = 0.003), cohort (F (1, 103.797); *p* < 0.001) and time*cohort effect (F (4, 42.122); *p* < 0.001). Cohort 1 (expl.) was different from cohort 2 (ctrl.) at W08 (*p* < 0.001), W12 (*p* = 0.007) and W16 (*p* < 0.001). Cohort 1 (expl.) improved over time with W08 [24.89 (6.00)%; *p* < 0.001] and W16 [29.53 (7.76)%; *p* = 0.039] MI-CAT(V) scores significantly lower than BSL [33.62 (6.89)%]. Whilst not significant, cohort 1 (expl.) also showed a decreased MI-CAT(V) score at W12 [29.94 (6.62)%; *p* = 0.057] when compared to BSL. The placebo group (cohort 2) degraded over time, with W16 [36.13 (9.23)%] MI-CAT(V) score increasing significantly when compared to BSL [31.27 (7.04)%; *p* = 0.012].

Finally, the individuals’ distribution as responders or non-responders confirmed the improvement observed in cohort 1 (Table 4). The responder rate was significantly higher at W08, 80% Tx effect vs. 14% placebo effect (*p* < 0.001), W12 33% Tx effect vs. 0% placebo effect (*p* = 0.042) and W16 40% Tx effect vs. 0% placebo effect (*p* = 0.018). At W08, the maximal intensity of difference between cohorts was of –30% in favour of cohort 1 (expl.). Overall, cohort 1 cats improved in their OA condition by 26% compared to BSL, whereas a majority of cats in cohort 2 remained constant or deteriorated throughout this study.

### 3.6. OA Severity Clusters

The OA cat population was distributed into two significantly distinct severity clusters (*p* < 0.001) using BSL MI-CAT(V) scores: moderate (8 cats in cohort 1 (expl.) and 9 cats in cohort 2 (ctrl.) with BSL scores of 21 to 35%) and severe OA (7 cats in cohort 1 (expl.) and 4 cats in cohort 2 (ctrl.) with BSL scores over 35%). One cat ID: F-045 obtained a mild BSL score of 18.18%, thus was excluded from the following analyses. As only two clusters resulted from this distribution, Mann–Whitney non-parametric tests were performed for cluster validation for BSL PGA, Stairs and NAM, as observed in MI-CAT(V).

Hindlimb PVF data normalised by stance time duration and BW showed a significant difference at BSL between moderate and severe OA clusters (*p* = 0.026), with worse weight-bearing in severe cats. Compared to BSL, only the moderate OA from cohort 2 (ctrl.) significantly deteriorated their hindlimb PVF values (at W12, *p* = 0.004; and W16, *p* = 0.002), while severe OA fluctuated over time.

Severe OA cats climbed fewer stairs up and down (*p* = 0.050) and performed slower (*p* = 0.033) than moderate OA cats at BSL. The linear mixed model analysis detected a cluster effect for both total number of stairs up and down (F (1, 25.077) = 5.995; *p* = 0.022) and time up per passage (F (1, 23.099) = 7.998; *p* = 0.010). In cohort 1 (expl.), the same degree of response was observed in both clusters for number of stairs up and down, and responder’s rate, but severe OA cats responded on time up, climbing up the stairs faster over time as soon as W4 (*p* = 0.003 at W4, *p* < 0.001 at W12 and *p* = 0.003 at W16, compared to BSL), while moderate OA cats remained stable over time (*p* > 0.191).

Severity of OA did not influence initial NAM at BSL (*p* = 0.452). In cohort 1 (expl.), moderate OA cats improved earlier, being different to severe OA cats at WE07 (*p* = 0.050). The difference was lost during subsequent WE timepoints, suggesting that severe OA made up for the delay. Moreover, from WE13, both clusters showed deterioration, without intercluster difference at WE14 (*p* = 0.249), WE15 (*p* = 0.082) and WE16 (*p* = 0.252), but moderate OA cats presented more remaining effect in NAM than severe OA cats during Ry.

Finally, despite the initial difference in BSL MI-CAT(V) (*p* < 0.001), no cluster effect was detected with the linear mixed model analysis on MI-CAT(V), and both clusters presented in cohort 1 (expl.) the same degree of response, with a slightly more elevated amplitude (by 3–7.5%) in the severe OA cluster. No intercluster differences were found, as well as in terms of responder rate or intensity of response.

## 4. Discussion

In this prospective, randomised, blinded, placebo-controlled study, the clinical efficacy and safety of an expl. therapeutic diet containing a blend of active nutraceuticals (EPA 35 mg/kg/day, DHA 51 mg/kg/day, curcuminoids 4 mg/kg/day, and hydrolysed collagen 208 mg/kg/day) designed to improve outcomes in cats with OA was compared to a placebo diet without the added nutraceuticals. The active ingredients and dosages chosen were based on this study of Lascelles et al. [14] and Comblain et al. [21]. Long-chain omega-3 fatty acids have been shown to be beneficial in the management of OA in both cat and dog research articles [14,50,51], as well as in a recent metanalysis [10]. Comblain et al. observed a reduction in pain indicators in OA dogs fed a diet with curcuminoids, hydrolysed collagen and green tea [21]. The collagen used in the present study was hydrolysed collagen, whose mode of action differs from undenatured type II collagen. The latter was proposed to induce some immune tolerance, turning off the up-regulated immune attack of cartilaginous collagen [52] when hydrolysed collagen is highly digestible and provides large amounts of glycine, proline, and hydroxyproline, amino acids essential for cartilage regeneration [53,54,55]. However, collagen peptides exert different biological effects that may be dependent on the peptide and amino acid profile of the hydrolysed collagen [56], supporting a high variability in response with the different peptide profiles [57]. Curcuminoids extracted from turmeric possess several biological activities, with particular interest in OA for their anti-inflammatory and antioxidant potency [25,26]. The main challenge of the curcumin form is its solubility and bioavailability. Cats consuming the expl. therapeutic diet showed significant improvements in four out of four validated outcomes, whereas cats in the placebo group worsened over time.

There currently is very little research available on the effects of nutraceuticals in feline OA: only five studies have been published, and they all used partially validated or non-validated subjective outcome assessment [14,50,51,58,59]. Three studies evaluated various nutraceuticals centred on omega-3s and green lipped mussels in a prospective, negatively controlled design without reaching intergroup statistical significance with either Client-Specific Outcome Measure (CSOM), or general quality of life (QoL) [14], or non-validated questionnaire completed by owners [50,51]. For the objective actimetry outcome, one clinical trial showed similar results to the present study, with activity counts increasing in the test diet and decreasing in the placebo diet, with intergroup differences [14]. Both remaining studies [58,59] tested for the efficacy of glucosamine-chondroitin sulfate using owner [58] and veterinary assessment [58,59] as well as owner-subjective questionnaires (CSOM, Feline Musculoskeletal Pain Index (FMPI) and QoL) and objective actimetry [59], without showing any Tx effect.

Subjective methods of evaluations allow bias to cloud results, and it becomes challenging to discern a substantial Tx effect from a change due to a caregiver’s anticipation of benefits. The caregiver placebo effect refers strictly to improved ratings of (subjective) outcome(s) in companion animals in the absence of improvement with objective measures [60]. In a secondary analysis of five analgesic trials conducted in feline OA, the authors of these studies calculated an average 68% [54–74%] placebo response with an owner-completed clinical metrology instrument, the CSOM [61]. The CSOM success was associated to actimetry success in only 36%, characterising a caregiver placebo effect [61]. Another owner-completed clinical metrology instrument, the FMPI, presented similar weaknesses to CSOM, with high placebo success that hid any detection of meloxicam Tx effect, and no relationship found with objective actimetry outcome [62,63]. Non-validated outcomes might not be reliable, specific, nor sensitive to Tx thus, the quality of the resulting findings might be questioned.

Due to the rigorous methodology and the use of multiple, previously validated, subjective and objective outcomes, the present study should warrant strong conclusions regarding the nutraceutical combination used in the management of feline OA. The four assessment outcomes (PGA, Stairs, NAM, MI-CAT(V)) were previously validated, being able to differentiate between healthy and diseased cats (specificity) [27,28,29], as well as to detect Tx(s) effect, such as those provided by NSAID (meloxicam or firocoxib), gabapentin, and tramadol (sensitivity) [27,29,30,31,32,33]. In the present study, the MI-CAT(V) showed good reliability between both BSLs (ICC = 0.893 [95% CI 0.786–0.948]) and moderate to good reliability between the raters presenting different levels of experience. This brings further validation to the MI-CAT(V) metrological instrument as it was previously only used by one single evaluator in prior concurrent validation studies [32,33]. Moreover, the clinical metrology instrument detected a significant Tx effect at W08, W12 and W16 which was corroborated by a major between-groups difference in responder rate, particularly at W08 (80% responders in cohort 1). This was facilitated by the low value in placebo rate being 14% at W08, and even 0% at W12 and W16. In addition, the results obtained from the four outcomes were consistent with each other, all showing strong evidence of Tx efficacy (Table 4), particularly the systematic intergroup difference in responder rate), which further illustrates the importance of validating assessment methods as it ensures the reliability, functionality, specificity and sensitivity of the outcome.

In a recent concurrent validation through testing for firocoxib analgesic efficacy [33], MI-CAT(V) clustering correlated to different degrees of alteration severity for NAM (mild > moderate, severe), and PGA (mild, moderate > severe). It was interesting to observe similar correlation in the present study: Biomechanical alterations (PGA, Stairs) were more pronounced initially in the severe (*N* = 11 cats with BSL scores over 35%) than in the moderate (*N* = 17 cats with BSL scores of 21 to 35%) cluster, and no difference was observed for spontaneous activity (NAM).

Moreover, mild OA presented a lower responsiveness (50%) to firocoxib Tx than moderate and severe OA (67% and 100%, respectively) [33]. Here, severe and moderate OA clusters presented same responsiveness to the expl. therapeutic diet with MI-CAT(V), reinforcing the results obtained with firocoxib [33]. In the present study, the severe cluster (*N* = 7 cats) responded to the therapeutic diet, more and faster on biomechanical assessments PGA and Stairs, than the moderate cluster (*N* = 8 cats). Interestingly, this corroborates the results obtained in a secondary analysis of PVF responsiveness to analgesic Txs in canine OA [64]. A dog with a more severe affliction was more susceptible to improving under current OA Txs. Specifically, with each increase in PVF by 1% BW (less lame) at BSL, the odds of being qualified as a responder to Tx decreased by 9% [64]. No difference in NAM responsiveness (approximately 75%) to firocoxib was previously observed for the three OA clusters [33]. This was similar in the present study, with the difference that considering the required delay of action for nutraceutical active ingredients, moderate OA cats improved one week earlier than severe OA cats and presented more remaining effects during the Ry period. This indicates that a severely afflicted OA cat would benefit similarly to a moderately afflicted OA cat from such therapeutic diet. management. This evidence is significant enough to consider a change in the current paradigm of feline OA.

Currently available pharmacological therapies, such as NSAIDs, are used by veterinarians as first-line Tx for feline OA management, despite their potential side effects (e.g., gastrointestinal erosions/ulcerations, nephrotoxicity, and hepatotoxicity). The present diet was devoid of any side effects and did not aggravate the pre-existing renal conditions of the three CKD-IRIS 1 cats (N = 3; diagnosed at BSL) that participated in this study, two being part of cohort 1 (expl.), and one being part of cohort 2 (ctrl.). This is especially relevant considering OA and CKD are often concomitant diseases in the feline geriatric population [65].

Additionally, many studies observed a negative rebound effect with NSAID withdrawal. This was particularly observed with low doses in meloxicam [27,29,58,63]. Moreover, Delsart et al. reported a negative rebound effect with firocoxib, but only in cats with a mild degree of OA (as well as cat ID: F-045 in the present study), the more severely affected OA cats maintaining their benefits on most tested outcomes [33]. Comparatively, the nutraceutical diet yielded a maximal NAM activity increase by 31% compared to BSL, one week after diet withdrawal (WE13); it maintained intergroup differences during the last two timepoints assessed (WE15 and WE16); the supplemented cohort (expl.) showed an overall 67% responder rate vs. 23% in the placebo-ctrl. group. Moreover, MI-CAT(V) maintained intergroup differences from W8 to W16, while hindlimb PVF and Stairs (total number of stairs climbed up and down) reached intergroup differences only at W16, well after Tx withdrawal. The 6-week delay in observing first changes in NAM and the remaining effects during Ry suggest that the active ingredients of the expl. therapeutic diet interfered with long-term neuropathological transformations associated with such OA chronic pain.

Some study limitations ought to be outlined: first, the clinical effectiveness is based on a combination of active ingredients. Bioavailability studies could be performed to understand the influence of each nutraceutical on clinical efficacy. Second, the MI-CAT(V) scoring was not always performed by all evaluators depending on timepoint evaluation which may have influenced the results, particularly at W12 when only one evaluator was present. Third, the insufficient sample size during clustering may have caused statistical type II error, thus a larger cat population should have been sampled. Fourth, quantitative sensory testing could have brought additional input on peripheral and central nervous sensitization caused by chronic OA pain. Future studies should compare this expl. therapeutic diet with NSAID or other analgesic and evaluate therapeutic efficacy and safety, at home with client-owned OA-afflicted cats.

## 5. Conclusions

Based on the improvement of the overall outcomes, this study demonstrated that the therapeutic diet containing a high level of EPA and DHA, with a specific ratio of EPA:DHA (0.69:1), turmeric extract and hydrolysed collagen, generated analgesic and functional effects in cats with painful, mobility-impairing OA. When compared with the placebo-ctrl. diet, cats who administered the expl. therapeutic diet showed increased PVF values, correlating to diminished lameness; improved performance of Stairs signifying lessened fatigue related to OA pain; increased NAM, suggesting improved spontaneous mobility and comfort; and reduced MI-CAT(V) scores, indicating a decrease in functional alterations. The MI-CAT(V) clinical metrology instrument was validated for inter-rater reliability, for minimal placebo effect (<15% responders) and for clustering of the OA level of impairment with absent OA (<9%), mild OA (9–20%), moderate OA (21–35%) and severe OA (>35%) degrees. The expl. therapeutic diet was shown to be equally effective in treating biomechanical OA pain in both moderately and severely affected cats, free of side effects. Finally, this diet was proven to yield a high rate of compliance to administration, which is of fundamental importance when treating chronic illnesses like OA.

## Figures and Tables

**Table 1:** Ingredients list (*in italic*, ingredients present in expl. diet only).

	Units	Control Diet	Experimental Diet
Protein	g/1000 Kcal	74.05	76.77
Fat	g/1000 Kcal	35.44	35.31
Crude fiber	g/1000 Kcal	13.75	15.52
Ash	g/1000 Kcal	14.28	14.45
*EPA*	g/1000 Kcal	0.00	0.72
*DHA*	g/1000 Kcal	0.00	1.04
Total n3 fatty acids	g/1000 Kcal	0.74	2.64
Total n6 fatty acids	g/1000 Kcal	8.32	7.93
Curcuminoids *	mg/1000 Kcal	0.00	83.00
*Hydrolysed collagen*	g/1000 Kcal	0.00	4.28
Energy NRC2006 CF	Kcal/kg as fed	3781	3738

Corn, chicken by-product meal, corn flour, wheat, wheat gluten, corn gluten meal, chicken fat, powdered cellulose, natural flavors, brewers rice, dried chicory root, vegetable oil, *hydrolysed collagen*, *marine microalgae oil*, *fish oil*, powdered psyllium seed husk, fructooligosaccharides, vitamins, potassium chloride, potassium citrate, L-lysine, DL-methionine, calcium carbonate, choline chloride, taurine, hydrolysed yeast, turmeric extract *, salt, rosemary extract, preserved with mixed tocopherols and citric acid, corn flour, and trace minerals. * Turmeric extract contained approximately 18% of curcuminoids.

**Table 2:** Summary of study events.

	BSL	Tx	Ry
Evaluation	W-03 to W-01	W00 to W12	W13 to W16
Clinical bioanalyses	At selection phase W-05	D00 * W08 W12	W16
Physical exam	At selection phase W-05	W12	W15
PGA	W-03 W-01	W04 W08 W12	W16
Stairs **	W-03	W04 W12	W16
NAM	WE-03 to WE-01	WE00 to WE12	WE13 to WE16
MI-CAT(V)	W-03 W-01	W04 W08 W12	W16

BSL, baseline; D, day; MI-CAT(V), Montreal Instrument for Cat Arthritis Testing, for Veterinarians; NAM, night-time actimetry monitoring; PGA, podobarometric gait analysis; Ry, recovery; Stairs, stairs assay compliance; Tx, treatment; W, week; WE, weekend. Timing of particular assessment was identical for both cohorts with ±3 days dependent on circumstance. General health observations were ongoing from W-04 to W16. * Blood sampling was performed at D00, just before the introduction of the new diet. ** The number of timepoints was reduced, compared to other outcomes, to not bore the cats with such straining assay.

**Table 3:** Summary of demographics—Mean (SD).

	Cohort 1 (Expl.) *N* = 15	*p*-Value Intragroup M:F Comparison	Cohort 2 (ctrl.) *N* = 14 *	*p*-Value Intragroup M:F Comparison	*p*-Value Intergroup
Number of cats (count)	M: 9 F: 6	0.715	M: 7 F: 7 *	0.715	0.715
Age (years)	M: 10 (1) F: 11 (1)	0.864	M: 12 (2) F: 11 (2)	1.000	0.172
Body weight (kg)	M: 5.53 (0.69) F: 4.29 (0.55)	0.008	M: 5.66 (0.46) F: 4.00 (0.39)	<0.001	0.652
Initial X-rays score	M: 8 (4) F: 9 (3)	1.000	M: 9 (5) F: 11 (5)	0.383	0.949
Initial MI-CAT(V) score (%)	M: 33.87 (10.09) F: 32.02 (8.53)	1.000	M: 29.70 (8.30) F: 33.16 (11.64)	0.383	0.505

Expl., experimental; ctrl., control; M, males; F, females; MI-CAT(V), Montreal Instrument for Cat Arthritis Testing, for Veterinarians. * Missing ID: F-046, she was excluded from this study.

**Table 4:** Responder rate [number (%)] in cohort 1 (expl.) and cohort 2 (ctrl.) over time, with maximal intensity of difference between both cohorts and intra-cohort 1.

		Responders [Number (%)]				Max. Intensity of Difference (%)	
Outcome	Cohort	W04	W08	W12	W16	Δ(expl.—ctrl.)	Δ(expl.—expl. BSL)
Hindlimb	1 (expl.)	6 (43%)	7 (50%)	9 (64%)	9 (64%) *	+26%	+10%
PVF	2 (ctrl.)	6 (43%)	4 (29%)	4 (29%)	2 (15%)	at W16	at W16
Stairs	1 (expl.)	4 (27%)	-	5 (33%)	13 (87%) *	+42%	+78%
	2 (ctrl.)	7 (50%)	-	5 (36%)	4 (31%)	at W16	at W16
NAM	1 (expl.)	Continuous weekly expressed follow-up			10 (67%) *	+35%	+31%
	2 (ctrl.)				3 (23%)	at W13	at W13
MI-CAT(V)	1 (expl.)	2 (13%)	12 (80%) *	5 (33%) *	6 (40%) *	−30%	−26%
	2 (ctrl.)	0 (0%)	2 (14%)	0 (0%)	0 (0%)	at W8	at W8

Cats in cohort 1 received the experimental (expl.) diet from week (W) 00 to W12, and cats in cohort 2 received the placebo-control (ctrl.) diet over the same period, where both cohorts received the ctrl. diet in baseline (BSL), and from W13 to W16. The treatment responder rate was calculated for each following outcome at four timepoints, W04, W08, W12 and W16: Most affected hindlimb peak vertical force (Hindlimb PVF, normalised by stance time duration and body weight; responder = increase in PVF vs. BSL), Stairs assay compliance (Stairs, number of stairs up and down; responder = increase in number vs. BSL), and Montreal Instrument for Cat Arthritis Testing, for Veterinarians [MI-CAT(V); responder = decrease in MI-CAT(V) by 15%+]. Night-time Actimetry Monitoring (NAM; overall assessment) was continuous over 20 weekends (3 BSLs, and 17 after initiating both diets administration); responder = positive or null slope after linear modelisation. The treatment responder rates of cohort 1 (expl. diet) and 2 (placebo-ctrl. diet) were compared. The intensity of difference between cohorts at every timepoint was determined and the maximal percentage of between-groups difference was summarised, as well the maximal intensity of difference vs. BSL for cohort 1. * Treatment responder rate is significantly different between cohorts (see text for exact *p*-values). BSL, baseline; Max., maximal.

## Data Availability

The datasets presented in this study can be found in an online repository: https://data.mendeley.com/datasets/p8n49ggknc/1, accessed on 13 August 2024.

## Supplementary Materials

The following supporting information can be downloaded at: https://www.mdpi.com/article/10.3390/ani14223298/s1, Appendix S1. Cats characteristics at inclusion. Appendix S2. MI-CAT(V) scoring sheet.
