# Supplementary material for: Efficacy and Safety of a Diet Enriched with EPA and DHA, Turmeric Extract and Hydrolysed Collagen in Management of Naturally Occurring Osteoarthritis in Cats: A Prospective, Randomised, Blinded, Placebo- and Time-Controlled Study
Source: Animals (Basel). 2024 Nov 16;14(22):3298. doi: 10.3390/ani14223298 (PMC11591455; doi:10.3390/ani14223298)
Supplement: Supplementary file 1 [file animals-14-03298-s001.zip › animals-3283749-supplementary.pdf]

APPENDIX S1 – Cats characteristics at inclusion

Cohort	Cat ID	Sex	Age (years)	BW (kg)	Number of affected joints	Total radiographic score (Sum of the score of each joint + number of affected joints)	MI-CAT(V) Mean BSL score (%)	BCS
1	F-001	F	10.5	3.83	2	10	35.76	6/9
1	F-004	F	10.5	4.90	2	4	34.55	5/9
1	F-027	F	11.5	4.39	6	12	27.88	5/9
1	F-014	F	9.5	4.85	2	10	40.00	5/9
1	F-016	F	11.5	4.22	3	6	21.21	5/9
1	F-020	F	9.5	3.52	2	10	32.73	5/9
1	F-002	M	9.5	6.03	1	2	28.48	5/9
1	F-003	M	12.5	6.66	4	10	27.27	5/9
1	F-005	M	9.5	5.71	2	10	34.55	5/9
1	F-011	M	8.5	5.51	2	4	26.06	5/9
1	F-012	M	10.5	5.49	3	10	32.12	5/9
1	F-019	M	10.5	4.60	2	12	25.45	5/9
1	F-021	M	10.5	4.38	4	14	49.70	5/9
1	F-022	M	10.5	5.30	1	2	40.00	5/9
1	F-024	M	11.5	5.22	3	11	41.21	5/9
2	F-025	F	12.5	4.59	2	8	46.06	5/9
2	F-026	F	10.5	4.28	2	6	30.91	5/9
2	F-031	F	9.5	3.79	4	10	33.94	5/9
2	F-036	F	14.5	4.31	7	14	33.33	5/9
2	F-037	F	12.5	3.78	6	21	26.67	5/9
2	F-041	F	10.5	3.55	3	7	47.27	5/9
2	F-045	F	9.5	3.73	3	10	13.94	5/9
2	F-008	M	9.5	6.22	1	3	30.30	5/9
2	F-028	M	14.2	5.80	2	8	39.39	5/9
2	F-030	M	9.5	5.14	6	19	19.39	5/9

2	F-035	M	13.5	5.41	2	10	39.39	5/9
2	F-017	M	12.2	5.17	2	8	27.27	5/9
2	F-043	M	10.5	5.62	2	4	25.45	5/9
2	F-044	M	11.5	6.28	2	8	26.67	5/9
2	F-046	F	11.5	3.88	4	10	23.03	5/9

Evaluator: ____________ Cat: ____________ Date: ____________

APPENDIX S2 MI-CAT(V) scoring sheet

Montreal Instrument for Cat Arthritis Testing, for Veterinarian use

Assessment Criteria / Degree of Alteration

1. Body Posture

	Normal	Mild	Severe/Obvious
A) Hindlimb placement	0	1	3
B) Forelimb flexion	0	1	3
C) Hindlimb flexion	0	1	3
D) Hindlimb weight bearing	0	1	3

2. Gait

A) Moves slowly with short uneven strides	0	1	3
B) Forelimb stiffness	0	1	3
C) Hindlimb stiffness	0	1	3
D) Forelimb range of motion	0	1	3
E) Hindlimb range of motion	0	1	3
F) Lateral spine movement	0	1	3

3. Obstacles

A) Overhead obstacle at elbow height of cat

					(no attempt)
Speed	0 (run)	1 (fast walk)	2 (slow walk)	3 (crawl)	4
Willingness	0 (immediate)	1 (1-2s pause)	2 (2-5s pause)	3 (>5s pause)	4
Scrape loudness	0 (no sound)	1 (slight scrape)	2 (moderate scrape)	3 (loud scrape)	4

[Assess when cat is chasing a highly desired object (e.g. treat) when the overhead obstacle]

B) Jumping from raised obstacle

	(barely perceptible)	(perceptible)	(loud)	(no attempt)
Front feet land heavily	0	1	3	4
Hind feet land heavily	0	1	3	4

4. Global distance exam

No mobility impairment — Worst possible mobility

1 — 2 — 3 — 4 — 5 — 6 — 7 — 8 — 9 — 10

MI-CAT(V) total score = sum of all items

1. Body Posture

Observe when cat standing still. DO NOT assess when cat moving or rubbing itself against objects or evaluators, playing or any other behaviours that may affect scoring. If one side (left or right) seems differently affected, score the more severely affected side.

1A) Hindlimb placement

Normal Score: 0

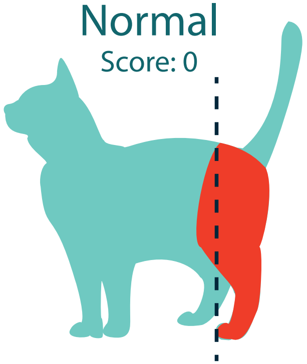

Hindlimbs straight

Mild Score: 1

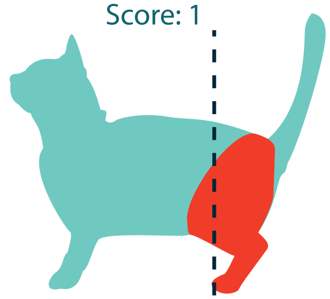

Hindlimbs bent and forward;
toes approaching middle of belly

Severe/obvious Score: 3

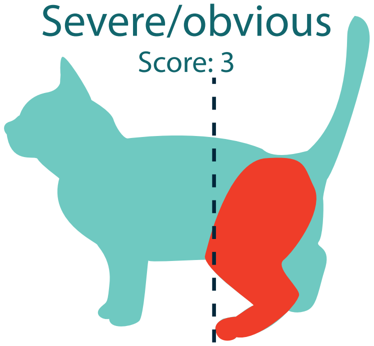

Hindlimbs bent and forward;
toes almost at middle of belly

1B) Forelimb flexion

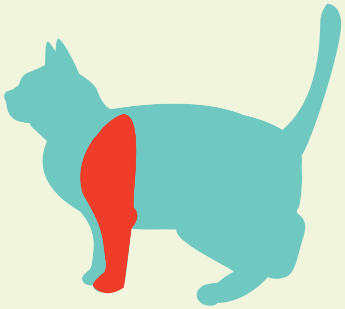

Forelimb straight

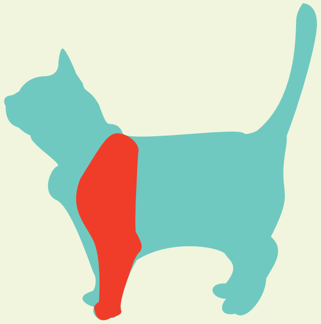

Forelimb slightly bent

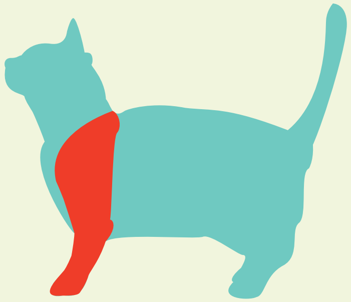

Forelimb obviously bent

1C) Hindlimb flexion

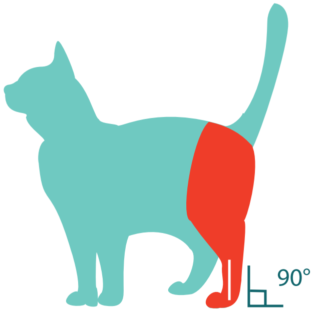

Hindlimbs **straight**
approx 90° to the floor

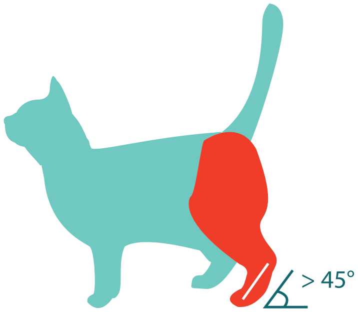

Hindlimbs **slightly bent**
approx > 45° formed between heel and floor

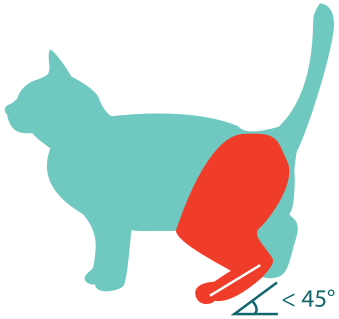

Hindlimbs bent, **heel close to ground**
approx. <45° formed between heel and floor

Designed by: VSY Leung - © GREPAQ 2020/03 - Update 2023/12

1. Body Posture

Observe when cat standing still. DO NOT assess when cat moving or rubbing itself against objects or evaluators, playing or any other behaviours that may affect scoring. If one side (left or right) seems differently affected, score the more severely affected side.

Normal
Score: 0

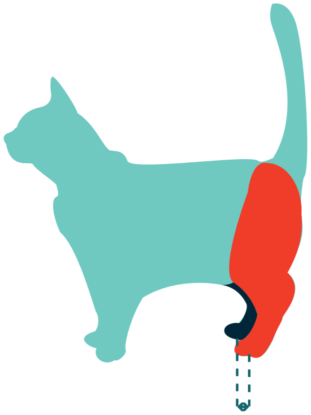

Feet side by side

Mild
Score: 1

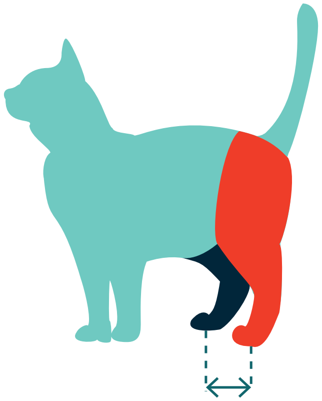

Feet moderately apart

Severe/obvious
Score: 3

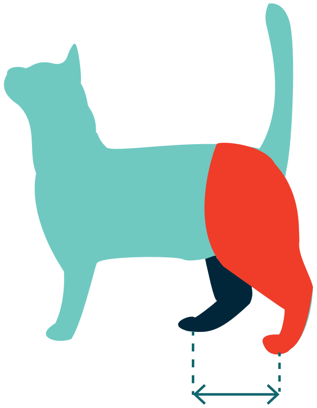

Feet widely apart

1D) Hindlimb weight bearing

-observe if body weight is evenly distributed on both hindfeet

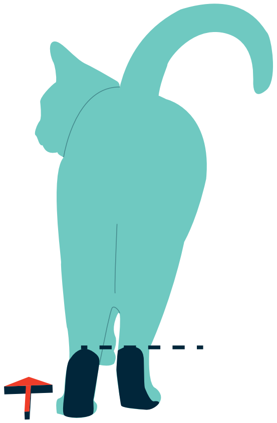

Feet **facing straight** forward
and
heels at similar heights

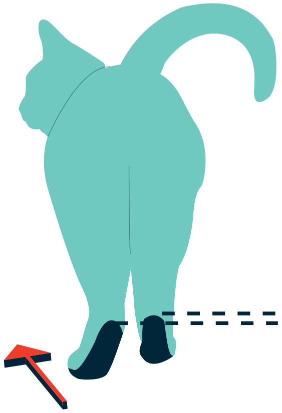

Feet **facing to side** slightly
and/or
heels at slightly different heights

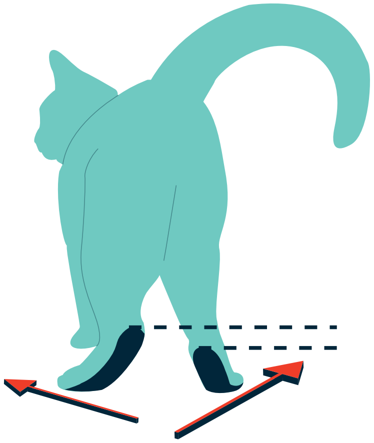

Feet **facing to side**
and/or
heels at different heights

Designed by: VSY Leung - © GREPAQ 2020/03 - Update 2023/12

2. Gait

Observe when cat is walking. DO NOT assess when cat is running (except for 2A), rubbing itself against objects or evaluators, playing or any other behaviours that may affect scoring. If one side (left or right) seems differently affected, score the more severely affected side.

	Normal<br>Score: 0	Mild<br>Score: 1	Severe/obvious<br>Score: 3

2A) Moves slowly with short uneven strides

-assess speed, quality of movement and overall willingness to move in general or chase desired object (*e.g.* treats)

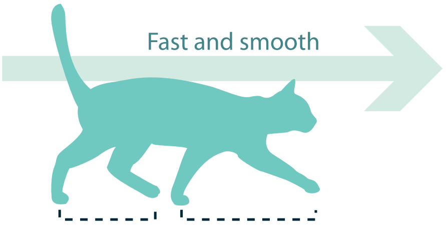

Cat runs or trots **quickly** with **long smooth strides**; **high motivation** to move

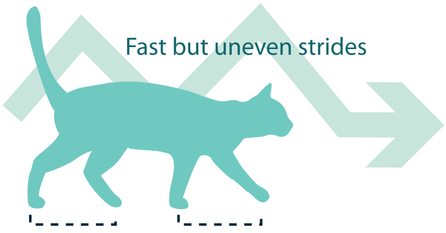

Cat runs or trots **quickly** with **short uneven strides**; **high motivation** to move

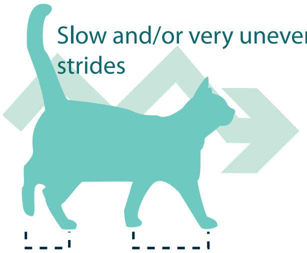

Cat walks **slowly or clumsily**; **low motivation** to move

2B) Forelimb stiffness

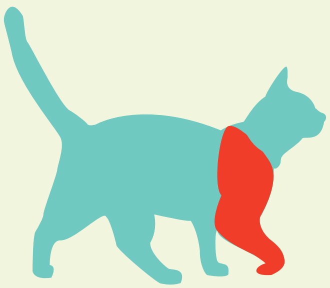

Forelimb muscles contract to lift paw and **metacarpal bends easily**

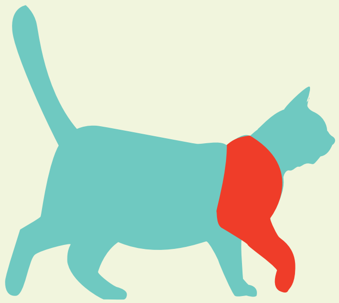

Forelimb muscles contract to lift paw and **metacarpal bends slightly**

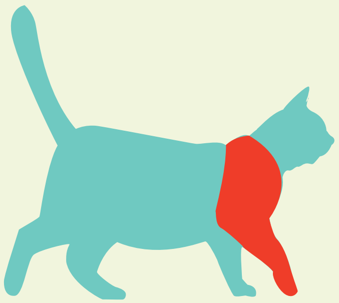

Forelimb muscles barely contract when lifting paw and **metacarpal barely bends**

2C) Hindlimb stiffness

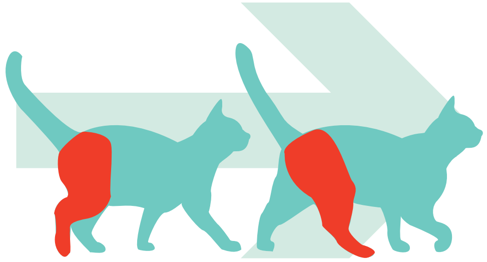

Hindlimb stretches forward when taking a step; **movement appears fluid**

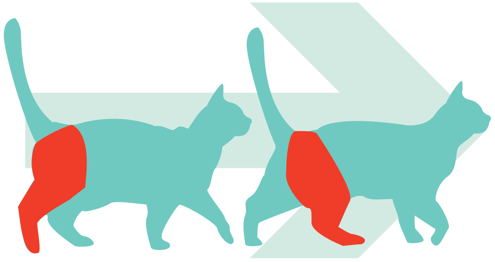

Hindlimb stretches forward slightly when taking a step; **movement appears slightly stiff**

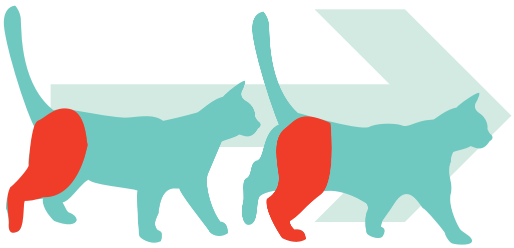

Angle between tibia and metatarsus stays unchanged; **cat appears to be waddling and movement is very stiff**

Designed by: VSY Leung - © GREPAQ 2020/03 - Update 2023/12

2. Gait

Observe when cat is walking. DO NOT assess when cat is running (except for 2A), rubbing itself against objects or evaluators, playing or any other behaviours that may affect scoring. If one side (left or right) seems differently affected, score the more severely affected side.

	Normal<br>Score: 0	Mild<br>Score: 1	Severe/obvious<br>Score: 3

2D) Forelimb range of motion

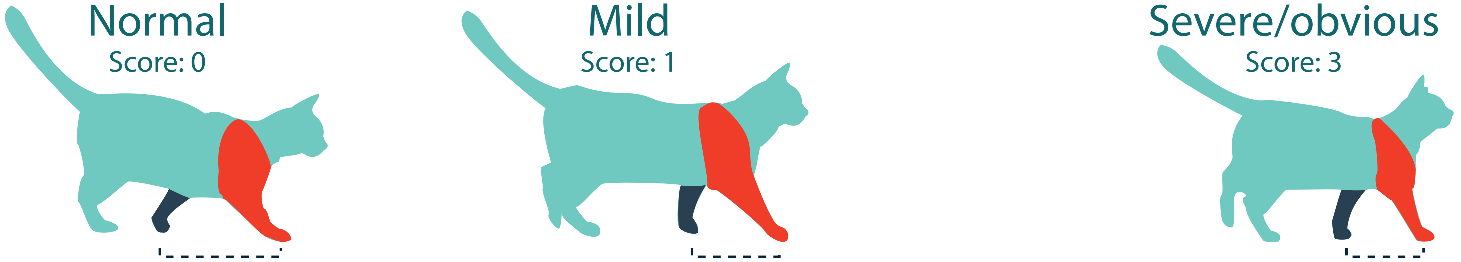

- Normal (Score: 0): **Forelimb stretches as far as possible** Wide distance between forepaws
- Mild (Score: 1): **Forelimb can stretch further** Smaller steps taken
- Severe/obvious (Score: 3): **Forelimb barely stretches forward** Small steps taken

2E) Hindlimb range of motion

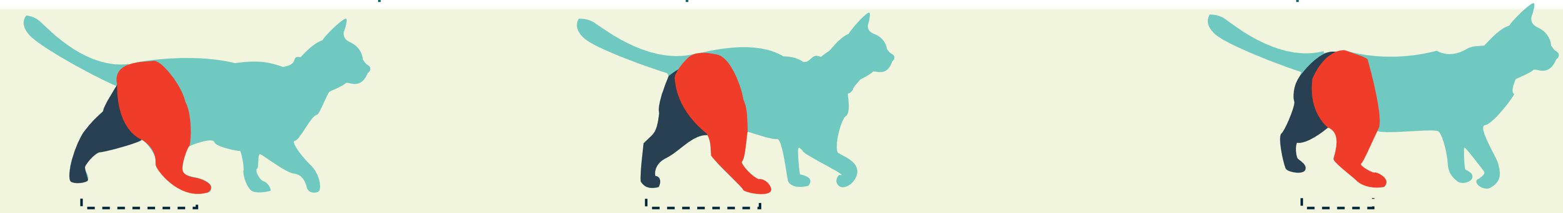

- Normal: Wide distance between hindpaws
- Mild: Wide distance between hindpaws
- Severe/obvious: Small steps taken Small distance between hindpaws

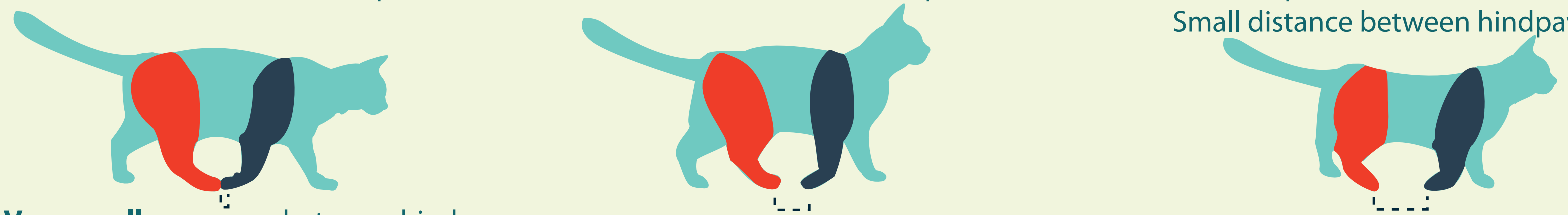

- Normal: **Very small or no gap** between hind- and forepaw, **hindpaw almost steps into place of forepaw**
- Mild: **Small noticeable gap** between hind- and forepaw, **hindpaw does not step into place of forepaw**
- Severe/obvious: **Wide and very noticeable gap** between hind- and forepaw, **hindpaw steps a large distance from where forepaw was**

2F) Lateral spine movement

-assess from behind while cat in motion

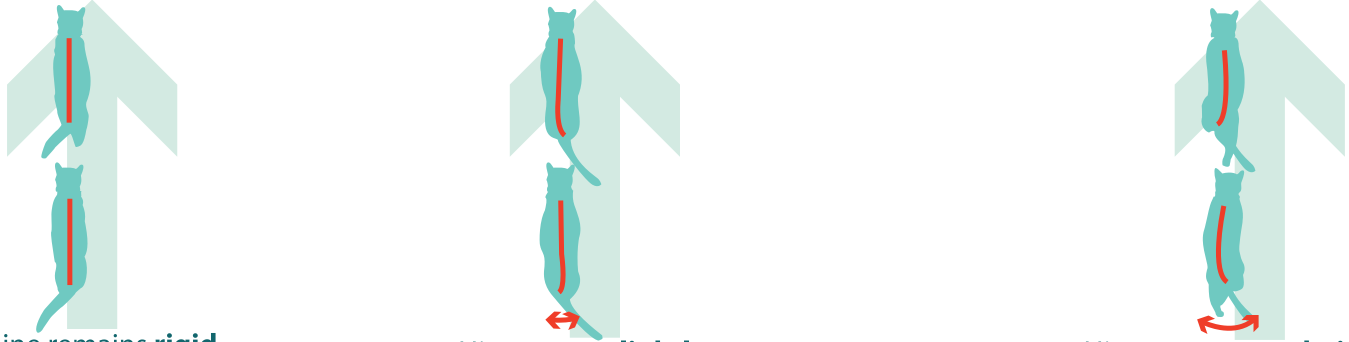

- Normal: Spine remains **rigid**
- Mild: Hips **sways slightly**
- Severe/obvious: Hip **sways very obviously**

Designed by: VSY Leung - © GREPAQ 2020/03 - Update 2023/12

3. Obstacles

Encourage cat to pass under overhead obstacle (cat's elbow height) and jump from table height (approx. 76 cm)
DO NOT physically push or pull cat by leash or collar or startle it. Use of treats or food is recommended.

3A) Difficulty passing under obstacle

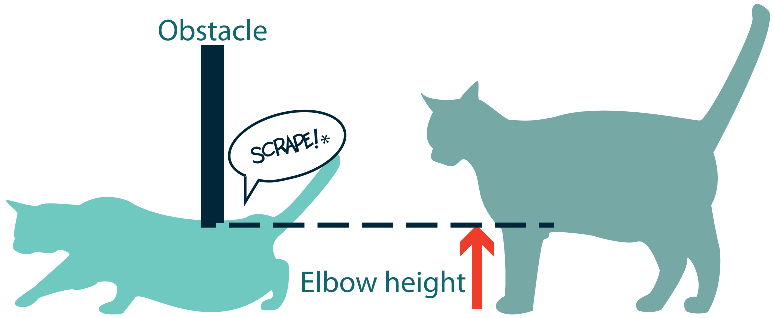

	Score 0	Score 1	Score 2	Score 3
**Speed:**	Run	Fast walk	Slow walk	Crawl
**Willingness:**	Immediate	1-2s pause	2-5s pause	>5s pause
**Scrape loudness*:**	No sound	Slight	Moderate	Loud

Right after throwing treat under overhead obstacle:

Speed = pace used to chase after treat
Willingness = reaction time to chase after treat and go under obstacle
Scrape loudness = sound of cat's back sccratching against obstacle as it goes under

Give score of 4 to each item if cat refuses to attempt

3B) Jumping from raised obstacle

*Assess loudness of landing for front and back feet:
not perceptible (score 0), perceptible (score 1), loud (score 3), did not try (score 4)

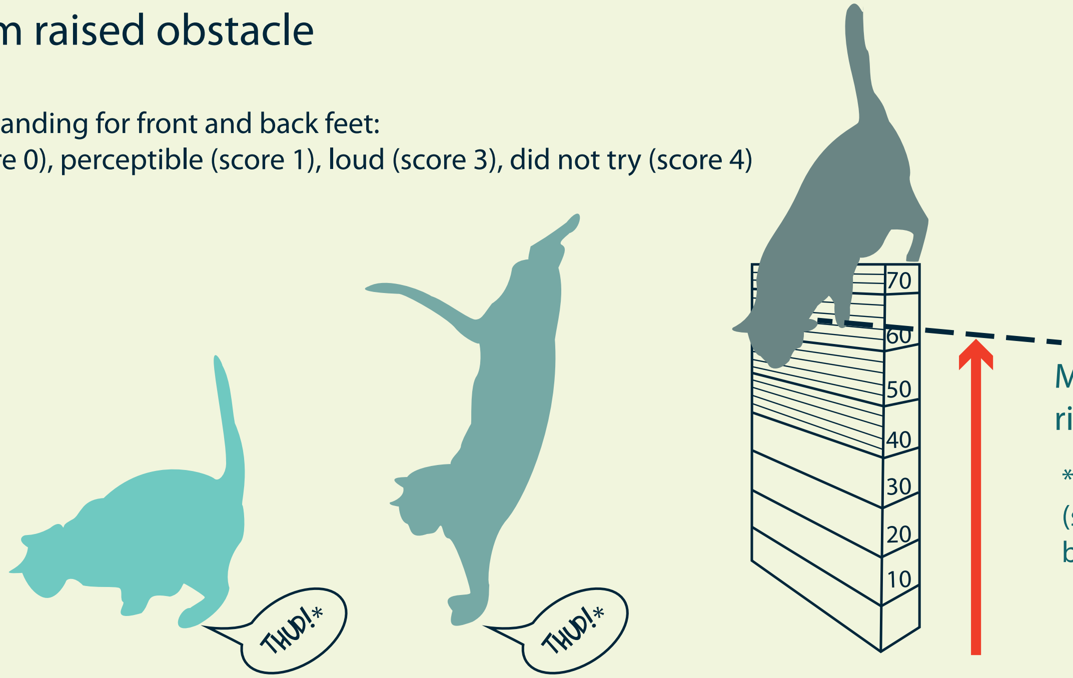

Measure height of cat's paw from ground right before jump**

**Recording and reviewing video footage (slow-motion feature) from a smart phone may be helpful.

Designed by: VSY Leung - © GREPAQ 2020/03 - Update 2023/12
